# Alcohol Use among Young Women in Kampala City: Comparing Self-Reported Survey Data with Presence of Urinary Ethyl Glucuronide Metabolite

**DOI:** 10.3390/ijerph21091256

**Published:** 2024-09-21

**Authors:** Monica H. Swahn, Jane Palmier, Rachel Culbreth, Godfrey S. Bbosa, Charles Natuhamya, Gideon Matovu, Rogers Kasirye

**Affiliations:** 1 Health Promotion and Physical Education, Wellstar College of Health and Human Services, Kennesaw State University, Kennesaw, GA 30144, USA; jpalmier@kennesaw.edu; 2 Toxicology Investigators Consortium, American College of Medical Toxicology, Phoenix, AZ 85028, USA; rachel.culbreth@acmt.net; 3 Department of Pharmacology & Therapeutics, Makerere University College of Health Sciences, P.O. Box 7072 Kampala, Uganda; godfossa@gmail.com; 4 Uganda Youth Development Link, P.O. Box 12659 Kampala, Uganda; natuhamyac@gmail.com (C.N.); giram010@gmail.com (G.M.); kasiryer@yahoo.com (R.K.)

**Keywords:** alcohol use, ethyl glucuronide, Uganda, substance use

## Abstract

This study sought to determine the level of concordance between self-reported alcohol use and the presence of its urinary ethyl glucuronide (EtG) metabolite in women living in urban Kampala. In 2023, we recruited 300 young women, ages 18 to 24 years, to participate in a prospective cohort study across three sites in urban Kampala (i.e., Banda, Bwaise, and Makindye) to examine the mechanistic pathways of mental illness. As part of the baseline assessment, participants were asked to complete a research assistant-administered survey and to provide a urine sample to screen for 16 different substances and/or their metabolites, including EtG. Overall, 58% (n = 174) reported to have ever consumed alcohol and 23% (n = 68) to have used it in the past month. Among the 300 women, 10% (n = 30) had EtG levels in their urine sample and of these, 40% (n = 12) reported to have never consumed alcohol, using a self-reported survey (*p* = 0.035). Recent alcohol use was relatively low among the women in this study. However, the discordance between self-reported alcohol use and the presence of EtG presents concerns about the accuracy of self-reported alcohol use. Additional research is needed to contextualize self-reported alcohol use, social desirability, and the implications for alcohol prevention and intervention strategies for young women in urban Kampala.

## 1. Introduction

Alcohol consumption is a significant contributor to various health problems, including liver disease, cardiovascular disorders, cancer, and mental health issues such as anxiety and depression [1,2,3,4]. As such, testing and screening for alcohol use plays a critical role in public health monitoring and epidemiological research. More specifically, monitoring alcohol consumption patterns and assessing the prevalence of recent alcohol use among different populations helps public health authorities identify at-risk groups, tailor interventions, and allocate resources effectively [5]. Furthermore, data from alcohol screening contribute to research on alcohol-related harm and inform evidence-based policies aimed at reducing alcohol-related morbidity and mortality [2]. Most of what is known about alcohol use patterns in populations is based on self-reports by individuals who are typically asked to disclose their own alcohol use behaviors. However, in some cases, such individuals may not disclose their alcohol use, nor may they recall its use accurately [6,7,8]. 

In fact, previous studies reported that self-reported alcohol consumption may not always align with alcohol use biomarker measurements like Phosphatidylethanol (PEth), EtG, and others [9,10]. This discrepancy between self-reported alcohol use versus the detection of metabolites of alcohol highlights the importance of utilizing objective measures of alcohol use biomarkers to accurately assess alcohol use, especially in populations where social desirability bias may impact self-reporting [8]. For instance, pregnant women in Uganda have been found to under report alcohol consumption, emphasizing the need for utilizing alcohol biomarkers testing to estimate their actual alcohol use [9,11,12]. 

While there are many different types of alcohol use metabolites that can be assessed [13,14,15], EtG has emerged as a relatively new and dependable alcohol use biomarker for evaluating recent alcohol use. EtG has favorable properties, including low cost and high sensitivity and specificity [16,17]. Moreover, the introduction of a commercially available enzyme immunoassay method for EtG analysis in urine has enhanced both the practicality and accessibility of EtG testing [18,19].

Research has demonstrated the efficacy of EtG testing for identifying alcohol consumption in liver disease patients awaiting transplantation, offering a longer detection window compared to breath alcohol testing, which is typically less than 24 h and subject to false positives [20,21,22]. The use of EtG as a biomarker for habitual alcohol consumption was also supported by population data, highlighting its potential as an objective marker in the general population [23]. EtG can be detected within 45 min of alcohol consumption, and small quantities of alcohol consumption can be detected using EtG testing 24 h later. For larger quantities of alcohol consumption, EtG can detect alcohol use up to 130 h after initial consumption [24]. Additionally, the assessment of alcohol consumption among healthcare workers, using EtG and ethyl sulfate (EtS) measurements in urine, has been effective in detecting alcohol exposure [25,26]. EtG has also been employed in professionals’ health programs to monitor alcohol abstinence, emphasizing its role as a direct alcohol biomarker [16,25,26,27]. 

While the association between alcohol use and adverse outcomes has been established across a variety of low-resource settings, specific high-risk populations, such as young women living in the slums of Kampala, warrant further investigation [28]. Alcohol use among women in Uganda is of significant concern, with research indicating a high prevalence of alcohol consumption at 78% [29]. Moreover, studies have noted a concerning increase in alcohol use among Ugandan women, raising worries about associated health risks and adverse effects across key developmental phases [30]. Alcohol consumption in early adulthood and adolescence may affect emotional regulation and impair stress responses according to preclinical animal models [31]. Additionally, sex differences in alcohol consumption in Uganda may be attributed to women’s hardships and involvement in commercial sex work, gender norms, gender physiological differences in ethanol metabolism, total body water, and variations in coping mechanisms among other factors [32,33,34]. Understanding the correlation between self-reported alcohol use and alcohol use measured using EtG can be critical to informing prevention initiatives and substance use interventions. Additionally, examining patterns of alcohol use and corresponding EtG results is critical for assessing alcohol-related risks and developing appropriate interventions for those with alcohol use disorders. This study aimed at assessing alcohol use among young women in Kampala and to compare self-reported survey data with the presence of urinary EtG to determine the accuracy of self-reporting of recent alcohol use for intervention planning and evaluations. We hypothesized that self-reported recent alcohol use would be discordant with urinary EtG results. 

## 2. Materials and Methods

In 2023, we recruited 300 young women aged 18 to 24 years, to participate in a prospective observational cohort study across three sites in Kampala city (i.e., Banda, Bwaise, and Makindye) to examine the mechanistic pathways of mental illness (TOPOWA study). This study was conducted in accordance with the ethical declaration of Helsinki. It was approved by the Uganda National Council of Science and Technology, UNCST (HS2959ES) and Makerere University School of Health Sciences (MaKSHS) Research and Ethics Committee (MAKSHSREC-2023-532). All participants provided written informed consent before taking part in this study. Participants also received remuneration for participating in the survey and for providing a urine sample. 

As part of the baseline assessment for the larger TOPOWA project, participants were asked to complete a research assistant-administered survey containing a broad range of measures pertaining to demographic and psychosocial characteristics and life experiences and to provide a urine sample to test for metabolites of 16 different substances and the EtG metabolite of ethanol. The study target population for the TOPOWA cohort study included the following: those who self-reported as female, 18–24 years of age, living within a radius of 2 KM from the Uganda Youth Development Link (UYDEL) vocational training centers, and those who had attained a minimum of primary five education level. Those who presented with self-reported pregnancy, significant intellectual disability or severe mental illness, or substance use requiring hospitalization were excluded from this study. As part of the baseline assessments, all participants were asked to complete a research assistant-administered survey and to provide a urine sample to test for 16 different substances, including the EtG metabolite of ethanol. 

### 2.1. Study Variables 

To validate self-reported alcohol and substance use, participants also consented to provide a urine sample to test 16 substances (i.e., EtG metabolite of ethanol, benzodiazepines, barbiturates, methadone, tetrahydrocannabinol [THC], K2 synthetic cannabinoid, cocaine (benzoylecgonine), amphetamines, phencyclidine [PCP], methamphetamine, buprenorphine, oxycodone, ecstasy, fentanyl, tramadol, and other opiates) in addition to contaminant adulteration tests. We selected a commercially available Multi-Drug Rapid Test Cup (Healgen Scientific Limited, Houston, TX, USA, Item number HCDOAEW-6165EFKTA3), a urine screening test that can be performed without the use of an instrument. The test utilizes antibodies to detect elevated levels of specific drug and or their metabolites in urine for 5 min using a cup with self-contained testing strips for all substances. The adulteration strip results including oxidants, pH, and specific gravity were read and compared with the manufacturer’s color chart. If the sample indicated positive results for adulteration, the participant was asked to provide another urine sample for re-testing. The results display only a control line when a substance is detected, and the absence of a substance is displayed with a positive, colored line. The urine drug screen results were reviewed and recorded at 5 min and then entered into REDCap using tablets. 

Self-reported alcohol use was assessed using the measures from the National Epidemiologic Survey on Alcohol and Related Conditions [35]. Participants were asked, “In your entire life, have you had at least 1 drink of any kind of alcohol, not counting small tastes or sips?” (Yes/No). All those who answered “Yes” with respect to lifetime alcohol use were asked a follow-up question about past-month alcohol use, “During the past 30 days, on how many days did you drink one or more drinks of an alcoholic beverage?” The responses were dichotomized and recorded to zero days of drinking in the past month versus 1 or more days of drinking.

### 2.2. Statistical Analysis 

EtG test results were reported as a binary variable (positive, negative). The participant’s response to ever drinking was binary (yes, no) while alcohol use in the past month was reported as a count variable (number of days in the past month that a participant had a drink), which was then recoded as a categorical variable (Didn’t drink [0 days], Had a drink [Some days], No response [Doesn’t know/remember or refused to answer], and N/A [Never had a drink their entire life]). Chi-square tests were used to assess the association between EtG test results and self-reported alcohol use. The statistical significance level was set a priori at *p* < 0.05. In this study, we examined whether there was a statistically significant difference between testing positive for alcohol use in the urine screening (EtG test results) and self-reported alcohol use in the past month or ever.

## 3. Results

Among the 300 participants included in this analysis, 52.0% (n = 156) were between 21 and 24 years of age, 66.0% (n = 198) had obtained at least some secondary education, and 62.0% (n = 186) had a biological child. Only 4.7% had neither of their parents alive, whereas 36.7% lived with their parents (Table 1). The results from the chi-square test indicated a statistically significant association between past-month alcohol use and having a child (*p* = 0.009).

Overall, the prevalence of ever drinking alcohol was 42.0% (n = 126) (Table 2). Of the women who reported ever drinking alcohol, 54.0% had a drink in the past month. Moreover, 14.3% reported no current alcohol use in the past month (former drinker), and 31.7% did not respond to the past-month alcohol-use question.

**Figure 1:**
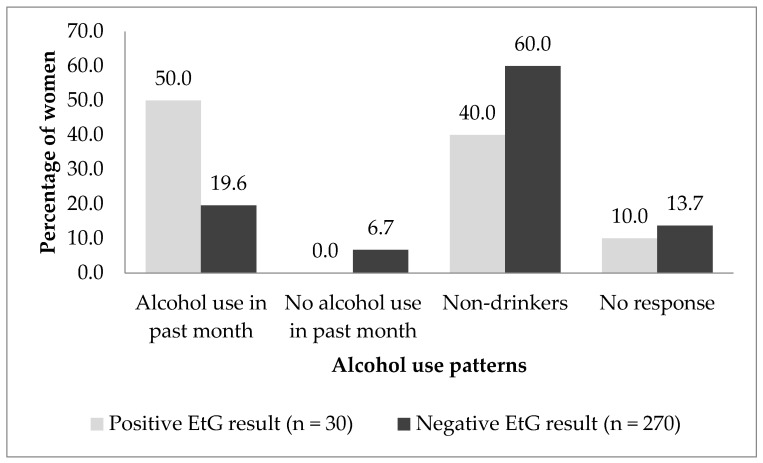
Association between self-reported past-month alcohol use and urinary EtG test result baseline assessment of the TOPOWA study of young women (Ages 18–24) (N = 300).

Among the 300 women, 10.0% (n = 30) had detectable EtG metabolites of ethanol in their urine sample, and among these, 40.0% (n = 12) reported never to have consumed alcohol (*p* = 0.035). An association was observed between self-reported alcohol use in the past month and EtG findings (*p* = 0.002), such that a higher percentage of women with positive EtG results also reported having a drink in the past month (50.0%) compared to those with negative EtG results (19.6%) (Figure 1).

**Figure 2:**
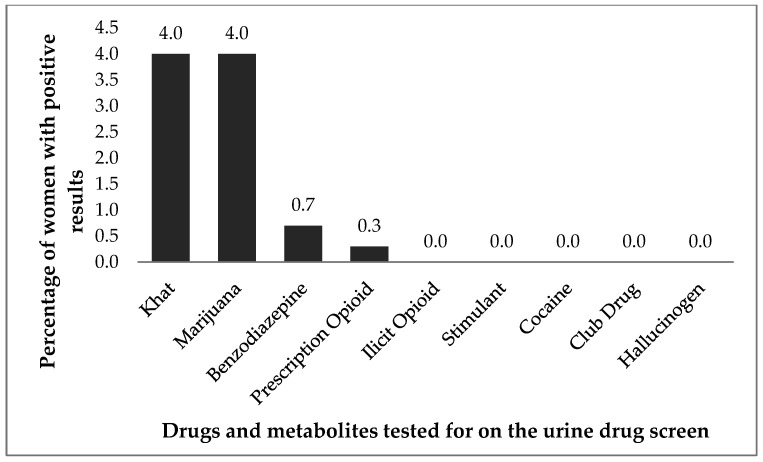
Urine drug test results among young women in the TOPOWA study, (N = 300).

Figure 2 displays the percentage of positive urine drug test results for drugs (excluding EtG). While Khat (4.0%) and marijuana (4.0%) were the most prevalent in the urine drug tests, the percentage of positive drug results were below 5% for the total sample. 

## 4. Discussion

This study assessed alcohol use among young women in Kampala city and compared self-reported survey data with the presence of urinary EtG metabolite of ethanol. Overall, 23.0% of participants reported alcohol consumption in the past 30 days, while 10.0% tested positive for the urinary EtG metabolite, indicating recent alcohol use. Notably, recent alcohol use, as indicated by EtG, was relatively low among the women in this study compared to previous research [9]. This discrepancy may be attributed to the narrow time frame captured by EtG, which detects metabolites typically within 72 h but can range between the past 24 h for minimal alcohol use and up to 5 days for heavy drinking [17,36,37]. The discrepancy between self-reported alcohol use and the presence of EtG in urine raises questions about the accuracy of self-reported data. Most importantly, in this study, 40% of those testing positive for EtG metabolites in urine reported no alcohol consumption in the past month. Previous research also noted similar discrepancies. A study by Bajunirwe and colleagues [9] found that 52.0% of women testing positive for Phosphatidylethanol (PEth) also reported no alcohol use in the prior 30 days. 

However, EtG has some limitations for cross reactivity and false positives. For example, hand sanitizer used by patients has triggered positive EtG urine results without prior ethanol consumption [38]. In patients tested for ethanol abstinence for liver transplantation, false positive EtG results were associated with bacterial metabolism, reduced kidney function, and other drug regimens [39]. However, these reasons behind EtG false positives are expected to be non-existent or low in our sample of young, healthy women.

Additional research is clearly needed to contextualize self-reported alcohol use and how social desirability and other factors may impact disclosing recent alcohol use for young women. We suspect that the 10.0% non-response rate among EtG-positive participants in this study could be attributed to social desirability bias, a major contributor to under reporting of alcohol use, particularly in settings where there is a perceived risk of negative consequences [40,41,42,43]. Social desirability has been associated with higher levels of stigma [44] and impression management among young people, leading to a tendency to portray socially acceptable behavior while denying socially deviant actions [7,45]. 

This study also found that 75.0% of women who had consumed alcohol in the past month had a biological child, compared to the 52.0% of non-drinkers who had a biological child. This is particularly concerning due to qualitative reports in Uganda of school-aged youth consuming alcohol given to them by their parents [46]. The intergenerational impacts of alcohol use in this population should be studied in the context of modifiable risk factors to prevent the cyclical effects of problematic alcohol use and inform secondary prevention interventions [47]. 

### Limitations

The findings in this study should be interpreted while keeping several limitations in mind. First, this study was not originally designed to assess the concordance between self-reported alcohol use and the presence of alcohol metabolites. As such, available measures did not capture the same specific time frame as the urine screening for metabolites. Our self-reported measure captured past month and lifetime prevalence (ever alcohol use). However, of most interest to us, is understanding the context of under reporting alcohol use in order for researchers and healthcare providers to improve or facilitate disclosure. As such, the presence of the alcohol metabolites quantifies to some degree the level of under reporting in the most conservative context. Second, we only tested for EtG in this study. While there are other metabolites that can be assessed with the same goal, EtG has emerged as an easily administered and low-cost self-contained rapid urine screen option that was suitable to this study and could be implemented by research assistants outside the clinical setting. Third, we did not have any follow-up measures to understand the context for omitting to disclose recent alcohol use. However, future research is urgently needed to understand the social desirability concerns as well as the stigma in self-reporting alcohol use. 

Additionally, 20% of those with negative EtG results reported having a drink in the past month. This discrepancy is likely related to limitations in the detection window of EtG and is subject to when the last time the participants drank alcohol. However, it should be noted that urinary EtG might not detect all recent alcohol use, which can be problematic for some heavy drinking patterns that are five or more days prior to the urine test. There are also issues with uncertain EtG results, which did not occur in this study but can lead to inconclusive results [48]. 

## 5. Conclusions

In conclusion, urinary EtG was a feasible and valuable biomarker for studying recent alcohol use in Kampala city, Uganda and potentially across other low-resource settings. Its high sensitivity and specificity, longer detection window, and reliability as a screening tool make it a valuable tool in assessing alcohol use, monitoring abstinence, and evaluating the effectiveness of interventions. In this study, we found the rapid urine screening test easy to administer, and it provided valuable information for us to consider how to best determine under reporting of alcohol use among the women in this study. The under reporting of recent alcohol use by the young women in this study needs to be considered in prevention and intervention programs targeting similar populations. This may be particularly relevant for any surveillance programs seeking to monitor trends in alcohol use patterns for young women or other initiatives seeking to assist public health authorities to identify at-risk groups, tailor interventions, and allocate resources effectively for alcohol prevention. 

## Figures and Tables

**Table 1:** Demographic characteristics and drinking status of participants in the baseline assessment of the TOPOWA study of young women (ages 18–24) (N = 300).

Characteristic	Alcohol Use in Past Month (n, %)		No Alcohol Use in Past Month (n, %)		Non-Drinkers (n, %)		No Response (n, %)		Total (n, %)		*p*-Value
Age											0.111
18–20	25	36.8	9	50.0	93	53.5	17	42.5	144	48.0	
21–24	43	63.2	9	50.0	81	46.6	23	57.5	156	52.0	
Education level											0.513
Primary or lower	28	41.2	7	38.9	52	29.9	15	37.5	102	34.0	
Some secondary	36	52.9	11	61.1	105	60.3	22	55.0	174	58.0	
Secondary or higher	4	5.9	0	0.0	17	9.8	3	7.5	24	8.0	
Had a biological child											0.009
Yes	51	75.0	13	72.2	94	54.0	28	70.0	186	62.0	
No	17	25.0	5	27.8	80	46.0	12	30.0	114	38.0	
Household size											0.090
<=4	44	64.7	6	33.3	99	56.9	20	50.0	169	56.3	
>4	24	35.3	12	66.7	75	43.1	20	50.0	131	43.7	
Status of parent(s)											0.852
Both parents alive	44	64.7	10	55.6	113	64.9	28	70.0	195	65.0	
One parent alive	21	30.9	6	33.3	54	31.0	10	25.0	91	30.3	
No parents alive	3	4.4	2	11.1	7	4.0	2	5.0	14	4.7	
Lives with parent(s)											0.872
Yes	24	35.3	7	38.9	66	37.9	13	32.5	110	36.7	
No	41	60.3	9	50.0	101	58.1	25	62.5	176	58.7	
Has no parent	3	4.4	2	11.1	7	4.0	2	5.0	14	4.7	
Generations in a household											0.055
One	14	20.6	6	33.3	23	13.2	2	5.0	45	15.0	
Two	30	44.1	6	33.3	97	55.8	21	52.5	154	51.3	
Three or more	24	35.3	6	33.3	54	31.0	17	42.5	101	33.7	

*p*: Pearson chi-square test *p*-value.

**Table 2:** Self-reported alcohol use versus urine EtG metabolite test results in the baseline assessment of the TOPOWA study of young women (ages 18–24) (N = 300).

Self-Reported Alcohol Use	Urinary EtG Metabolite Test Result		
	Positive (n = 30)	Negative (n = 270)	Total (N = 300)
Ever had a drink		N (%)	
Yes	18 (60.0)	108 (40.0)	126 (42.0)
No	12 (40.0)	162 (60.0)	174 (58.0)
*p* = 0.035			
Alcohol use in the past month			
Didn’t drink	0 (0.0)	18 (6.7)	18 (6.0)
Had a drink	15 (50.0)	53 (19.6)	68 (22.7)
No response	3 (10.0)	37 (13.7)	40 (13.3)
Non-drinkers	12 (40.0)	162 (60.0)	174 (58.0)
*p* = 0.002			

## Data Availability

The data may be available to interested collaborators upon request to the PI, Dr. Monica H. Swahn.
